# Repetitive somatosensory stimulation shrinks the body image

**DOI:** 10.1098/rspb.2025.1714

**Published:** 2025-10-29

**Authors:** Malika Azaroual-Sentucq, Silvia Macchione, Luke Miller, Eric Koun, Romeo Salemme, Matthew R. Longo, Alessandro Farnè, Dollyane Muret

**Affiliations:** ^1^ Integrative Multisensory Perception Action and Cognition team INSERM U1028, CNRS UMR5292, Université UCBL Lyon1, Centre de Recherche en Neurosciences de Lyon, Bron 69500, France; ^2^ Radboud University Donders Centre for Cognition, Nijmegen 6525 GD, The Netherlands; ^3^ Department of Psychological Sciences, Birkbeck, University of London, London WC1E 7HX, UK; ^4^ Department of Neuro-immersion, Hospices Civils de Lyon, Bron, Auvergne-Rhône-Alpes 69500, France; ^5^ Integrative Neuroscience and Cognition Center (INCC), CNRS UMR 8002, Université Paris Cité, Paris 75006, France

**Keywords:** mental body representations, tactile localization, somatosensory plasticity, touch

## Abstract

Current models of mental body representations (MBRs) indicate that tactile inputs feed some of them for different functions, implying that altering tactile inputs may affect mental body representations differently. Here, we tested this hypothesis by leveraging repetitive somatosensory stimulation (RSS), known to improve tactile perception by modulating primary somatosensory cortex (SI) activity, and measured its effects over the *body image*, *body model* and *superficial schema* in a randomized sham-controlled, double-blind crossover study. Results show that repetitive somatosensory stimulation affected the *body image*, participants perceiving their finger size as being smaller after repetitive somatosensory stimulation. While previous work showed an increase in finger size perception after tactile anaesthesia, these findings reveal that tactile inputs can diametrically modulate the *body image*. In contrast, repetitive somatosensory stimulation did not seem to alter the *body model* or *superficial schema*. In addition, we report a novel mislocalization pattern, with a bias towards the middle finger in the distal phalanges that reverses towards the thumb in the proximal phalanx, enriching the known distortions of the *superficial schema*. Overall, these findings provide novel insights into the functional organization of mental body representations and their relationships with somatosensory information. Reducing the perceived body size through repetitive somatosensory stimulation could be useful in helping treat *body image* disturbances.

## 1. Introduction

Mental body representations (MBRs) are critical for fundamental sensory abilities, such as localizing touches on our body surface, estimating body part size and maintaining a coherent sense of bodily self [1–3]. Several models were proposed, displaying different MBRs articulated in different ways. While some models consider the existence of two MBRs (see [4] for a review), some more recent models advocate for many more [2,3,5], each related to specific sensorimotor functions. Here, we focus on the somatosensory aspects of MBRs, overlooking their motor (involved in guiding actions; [6]), affective (feelings, (dis)satisfaction about the body; [7]) or semantic aspects (conceptual knowledge about body parts; [8]). The MBRs thought to be primarily involved in and dependent upon somatosensory processing are the *body image* [2,6], *body model* and *superficial schema* [2,9]. Although these MBRs were separately described to serve distinct purposes, they are acknowledged to interact (see [3,10] for recent reviews).

The *body image* is considered a conscious representation of the body, allowing, for instance, to estimate one’s hand’s shape and size. It was shown to be relatively accurate when assessed through depictive tasks, such as the template matching task [11,12], but quite distorted when assessed through more indirect metric methods, for example, requiring the comparison of body parts to physical lengths [13,14], or under the influence of multisensory illusions (e.g. [15,16]). The *body model* is thought to encode metric properties of the body, such as distances between joints, which allows us to judge metric properties like the distance perceived between two tactile stimuli [12]. Conversely, the *superficial schema* is thought to encode spatial information used to locate tactile stimuli on the body surface [5,9]. Both the *body model* and the *superficial schema* relate to the spatial properties of touch. Whether inherent to the representations or a product of near-optimal Bayesian integration of somatosensory inputs [17], perceptual distortions are consistently observed when assessing both of these MBRs. Indeed, Longo & Haggard [11,12] observed that the hand is perceived wider and the fingers shorter when using indirect measures of hand size (e.g. the tactile distance perception and landmark localization tasks), indicating that the *body model* may retain part of primary somatosensory cortex (SI) homuncular distortions. Similarly, using a tactile localization task on the hand’s dorsum, Mancini *et al*. [18] reported distal biases towards fingertips and thumb, indicating that the *superficial schema* may also be distorted. While these three tasks capture only some aspects of MBRs, they tap into complementary perceptual aspects of MBRs involving somatosensory processing.

These MBRs are built and maintained by multisensory inputs, including somatosensory [19], visual [20,21] and auditory inputs [22]. But evidence of the importance of somatosensory inputs in MBRs came from developmental studies [20], as well as studies involving multisensory stimulations [15,16]. While it is widely accepted that somatosensory processes contribute to building and maintaining MBRs through constant updates from the primary somatosensory cortex (SI; [1]), whether different MBRs are equally or differentially fed by somatosensory processing remains an open question. To start addressing this question, pioneering studies investigated the effects of modulating somatosensory processing on a single MBR. For instance, seminal work by Gandevia & Phegan [23] on *body image* revealed that the body parts whose tactile inputs were temporarily reduced by anaesthesia were perceived as bigger. More recently, Giurgola *et al*. [24] showed that interfering with the activity of SI hand representation via repetitive transcranial magnetic stimulation (rTMS) resulted in an overestimation of hand size. Conversely, high-frequency tactile stimulation reduced the perceived size of the stimulated hand [25]. Thus, perception of body part size via the *body image* seems to be modified when tactile processing is altered. Moreover, TMS over the SI hand representation impairs tactile identification of stimulated fingers [26], suggesting that interfering with SI function is also detrimental to the *superficial schema*.

While these studies indicate a tight link between somatosensory processes and MBRs, they typically investigated only one or two MBRs. To gain deeper insights into this link, we assessed the potential impact that altering somatosensory processes may have on the three above-mentioned MBRs. To this aim, we leveraged the properties of repetitive somatosensory stimulation (RSS), known to temporarily improve tactile perception by modulating SI activity [27]. RSS consists of the passive stimulation of a body part to induce synchronized neuronal activations in the corresponding SI representation, resulting in transient plasticity (see [27] for review). Here, we used RSS to temporarily alter somatosensory processes by increasing tactile inputs and investigate its effect on the three aforementioned MBRs, as assessed through three well-established paradigms: (i) the template matching task (TMT) measuring the perceived size of the finger (involving the *body image* [11,13,23]), (ii) the tactile distance judgement task (TDJT) assessing the distance between two tactile stimuli (involving the *body model*; [12,28]) and (iii) the tactile localization task (TLT) measuring the localization of tactile stimuli (involving the *superficial schema*; [18]). Since they are based on the same multisensory (somatosensory and visual) information, these three tasks provide a consistent basis for MBR comparison. While it is important to acknowledge that RSS represents only one way to alter somatosensory processing and that these three tasks provide only a partial picture of something as complex as MBRs, we posited that if the representations measured by these three tasks are similarly sustained by somatosensory processing, altering such activity through RSS should affect them all. Alternatively, if they bear different (direct or indirect) relationships with these somatosensory processes, some of them should be affected differently.

We found that RSS alters participants’ *body image* (TMT), without significantly modifying the other MBRs/tasks. While we refrain from drawing definitive conclusions, the present findings suggest that these MBRs may not be all equally dependent on somatosensory processes. Moreover, we found that increased tactile inputs (RSS) to the finger *reduces* the perceived finger size. This result, opposite to Gandevia & Phegan’s original report [23] obtained following the reduction of tactile inputs (anaesthesia), suggests that the *body image* is sensitive to somatosensory modulation in both directions. These findings allow us to refine current theoretical models with a deeper understanding of MBRs’ interrelationship. In addition, they open new avenues for the diagnosis and treatment of clinical conditions whereby the *body image* is altered.

## 2. Methods

### (a) Participants

We included 33 healthy adults (27 women; mean age ± s.d.: 22.8 ± 3.4 years), as determined by a power analysis using G*Power 3.1 [29] based on the work on TMT [30] and TDJT [22,31,32], showing medium (Cohen’s *d* = 0.5) to large (*d* = 0.8) effect sizes with sample sizes ranging between 8 and 20. Sixteen to 34 participants were required to detect a large-to-medium effect with 80% power.

Participants were right-handed (Edinburgh handedness inventory [33], average score ± s.d.: 85.2 ± 15.6), with no neurological or psychiatric disease, and no history of injuries at the right index finger (rD2). They gave written informed consent before participating and received compensation at the end of the study. Procedures were approved by the French ethics committee (CPP SUD EST IV n. ID RCB: 2010-A01180-39).

### (b) Experimental timeline

**Figure 1.**
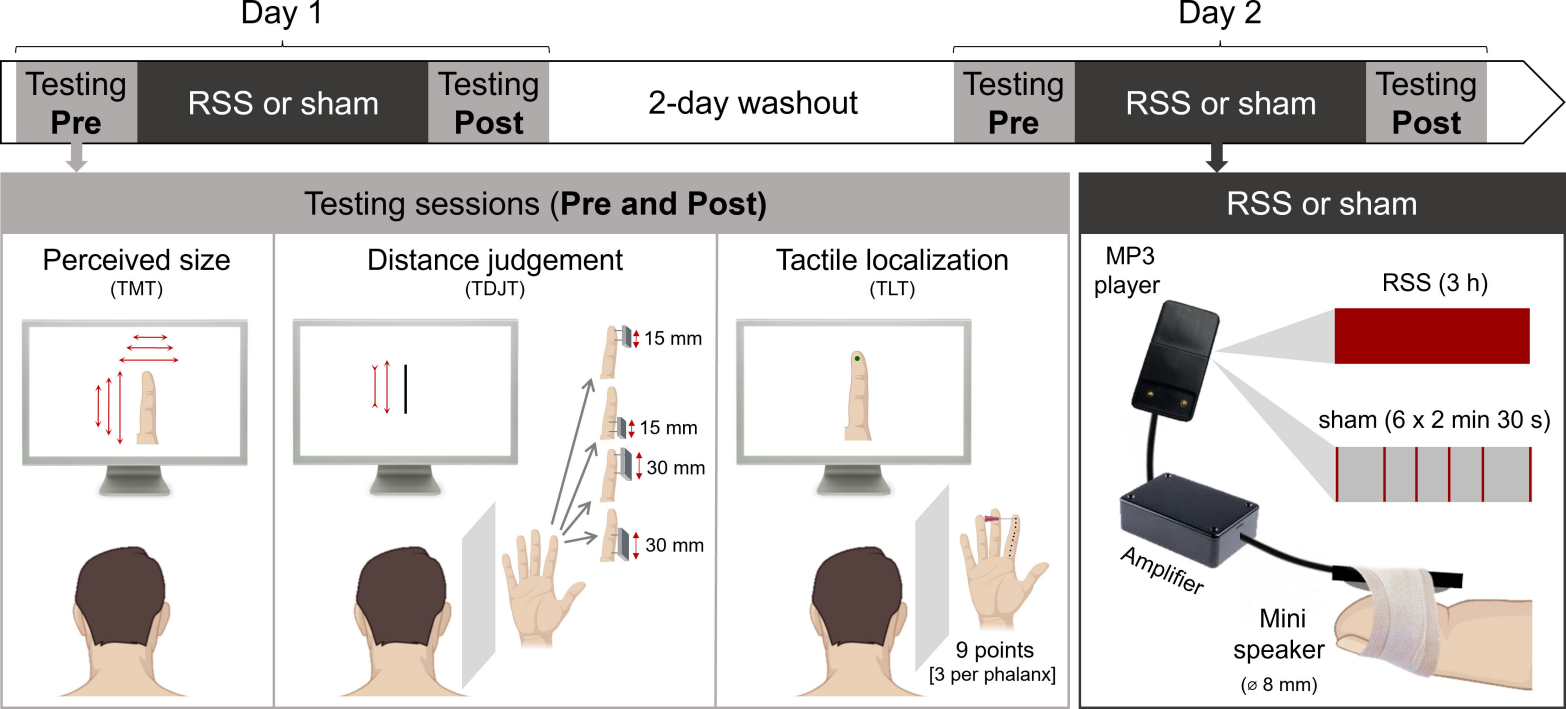
Experimental timeline and depiction of the tasks and interventions used. Participants received sham and RSS on two different days (counterbalanced order). Before and after each intervention, perceived finger size, tactile distance judgement and tactile localization were assessed through the template matching task (TMT), the tactile distance judgement task (TDJT) and the tactile localization task (TLT), respectively, in addition to the 2PDT (not shown).

A randomized, double-blind, sham-controlled design was used (figure 1). All participants received RSS and sham interventions on the rD2 on two different days separated by a 2-day washout period, RSS effects on tactile perception being known to last up to 6 h [34]. Half of them received RSS first, and the other half received sham first, each participant being randomly assigned to either group. No order effect was found for any of the tasks (all *t *≤ 6.10, *p* ≥ 0.06; see electronic supplementary material, table S1 for details). The effects of these interventions (RSS/sham) on rD2 MBRs were investigated through pre- and post-testing sessions, including the TMT, TDJT and TLT delivered in a counterbalanced order. To verify RSS efficacy, tactile discrimination was also assessed before and after interventions through the two-point discrimination task (2PDT). In all tasks, participants were instructed to focus on their rD2 sensations and respond as accurately as possible, with no time limits or feedback about their performance (see electronic supplementary material for details).

Before the experiment, participants’ rD2 was high-resolution colour scanned, and the image was resized to match the real size of their finger using Photoshop, before being used in the TMT and TLT. The three tasks were implemented using MATLAB (MathWorks, v.2015b).

### (c) Interventions (repetitive somatosensory stimulation and sham)

The intervention protocols consisted of a 3 h task-free mechanical stimulation on the rD2. A small (8 mm diameter) mini loudspeaker (LSM-S20K, Ekulit) controlled by an MP3 player (Lenco Xemio240 4 GB) was taped to the rD2 (figure 1). In the RSS protocol, this mini loudspeaker delivered brief (10 ms) supra-threshold tactile stimuli for 3 h, with inter-stimulus intervals ranging from 100 to 3000 ms and following a Poisson distribution (average stimulation frequency of 1 Hz). The sham protocol consisted of 15 min of tactile stimulation distributed across the 3 h (six blocks of 2.5 min each). Within each block, the mini loudspeaker delivered tactile stimuli with the same frequency and distribution as during RSS.

### (d) Two-point discrimination task

Tactile acuity on the rD2 was assessed using the 2PDT with a well-established procedure [27]. Eight probes were presented on the volar surface of the fingertip: one with a single tip and seven with two tips separated by various distances (0.7, 1, 1.3, 1.6, 1.9, 2.2, 2.5 mm). Each probe was tested eight times in a pseudo-randomized order (64 trials per session), with the tips aligned to the longitudinal axis of the finger. Participants were blindfolded and asked to indicate whether they perceived ‘one’ or ‘two’ tips at each trial with the specific instruction of saying ‘two’ only when the tips were clearly distinguishable.

The average of the verbal responses (‘one’ or ‘two’) was computed, and the percentage of ‘two’ responses was plotted as a function of the distance between probes. The psychometric function was fitted with a binary logistic regression (Statistica Tibco, v.13.3), and the point of subjective equality (PSE) was determined as the threshold at which participants were at chance level for each session (pre and post) and intervention (sham and RSS).

### (e) Template matching task

Participants were seated in front of a computer screen (55 cm away), tilted at 30° above the horizontal plane, with their left hand on a keyboard and their right hand hidden under the table, open with their palm facing upwards. At each trial, an image of their real rD2 (real size, larger or smaller) was displayed on the screen, and they were asked to judge whether it was smaller or larger than their finger by pressing the corresponding keyboard buttons (+ or −).

The stimuli were as follows: four enlarged, four reduced (with uniform area distortions of ± 3%, ± 6%, ± 9%, ± 12% relative to the actual finger size), and one real-sized image of the finger. Each stimulus was presented 12 times in randomized order for a total of 108 trials. The percentage of responses corresponding to ‘image perceived larger’ was plotted as a function of finger image distortion, and a psychometric function was fitted with a binary logistic regression. From these fitted data, the PSE, at which participants perceived the image as big as their finger, was determined as the distortion threshold at which they were at chance level.

### (f) Tactile distance judgement task

Participants were seated in front of a computer screen (55 cm away), with their unseen right hand lying supine on the table behind an occluding board. The experimenter touched the volar surface of their rD2 with two wooden rods simultaneously applied along the longitudinal axis, either within a single phalanx (i.e. rods spaced by 15 mm) or across two adjacent phalanges (i.e. rods spaced by 30 mm). For each distance, the rods were applied (for approximately 1 s) starting from two different locations: the base or the tip of the finger (figure 1, middle lower panel). Each condition was repeated 10 times in a pseudo-randomized order, for a total of 40 trials. Participants were instructed to assess the tactile distance between the two rods by adjusting the length of a bar on the screen (+ and – buttons) to match the perceived distance. The estimated bar length was recorded and averaged for each session (pre, post), intervention (sham, RSS) and position (base, tip).

### (g) Tactile localization task

Participants were seated in front of a computer screen (as above). The experimenter touched the volar surface of their rD2 with a plastic von Frey monofilament of 5 g, at one of nine different locations on the longitudinal midline of the finger, with three positions per phalanx: one-quarter, one-half and three-quarters of the length of each phalanx. Each location was touched 10 times in a pseudo-randomized order, for a total of 90 trials. The real-sized image of their own finger was displayed on the screen, and they were asked to indicate the location of the perceived touch on that image. To do so, participants moved with their left hand a cursor on the screen to the desired location and validated their choice by pressing a button.

Both the judged (J) and real (R) locations were recorded as *x* and *y* coordinates of the picture displayed on the screen. The origin of the coordinate system was centred on each of the real locations, with the *y*-axis representing the longitudinal (proximo-distal) axis of the finger and the *x*-axis the bottom medio-lateral (ulnar-radial) axis of the finger. After normalizing the coordinates to each participant’s finger length, three measures were calculated for each of the nine locations (see electronic supplementary material, figure S1): (i) the Euclidean distance between the J and R locations: $\Delta_{JR}=\sqrt{{\left(x_{J}-x_{R}\right)}^{2}+{\left(y_{J}-y_{R}\right)}^{2}\;}$, (ii) the polar angle between the JR vector and the *x*-axis at the R location: ${Ө}_{JR}=a\tan2\left(\frac{y_{J}-y_{R}}{x_{J}-x_{R}}\right)$, (iii) 95% confidence ellipses of the judged locations (the mean judged location (x̄_J_, ȳ_J_) being the centre of the ellipse). The mean J–R distance (magnitude) and the mean J–R angle (direction) were used as a measure of constant error of tactile localization, while the mean area of the confidence ellipse was considered a measure of the variable error.

### (h) Statistical analysis

Data were collected through MATLAB (MathWorks, v.2015b) and are accessible through Dryad (doi: https://doi.org/10.5061/dryad.t4b8gtj9d [35]). Data were missing for two participants in the TLT (for technical reasons), and two participants were removed from the analysis of the TMT, given the impossibility of fitting the data with a binary logistic regression. The number of participants included in each task is as follows: *n* = 33 in the 2PDT and TDJT, *n* = 32 in the variable error of TLT and *n* = 31 in the constant error of TLT and in the TMT, with 30 participants common to the four tasks. Outliers were defined as falling outside 3 s.d. around the average. First, in tasks containing single trials (TDJT and TLT), outlier trials were inspected (intra-subjects) but no outlier was found. In the TLT, a few missed trials were removed, representing 0.03, 0.21, 0.35 and 0.14% of the data in the pre-sham, post-sham, pre-RSS and post-RSS protocols, respectively. After trial removal, a minimum of 7/10 trials were left for each condition. Then, in all tasks, outliers were identified at the group level (inter-subjects). When present, statistical analyses are reported in the results section, both including and excluding these outliers, as this did not change the findings. Results in the text are expressed as mean ± s.e.m.

Linear mixed-effects models (LMMs) were applied to the data. For all tasks, the models included the factors session (pre and post) and intervention (sham and RSS) and their interaction as fixed effects. For the TDJT and TLT, additional fixed-effect factors were included: position (finger tip and base) and distance (15 and 30 mm) for the TDJT, and phalanx (proximal, middle, distal) and position (n°1, n°2 or n°3 at each phalanx) for the TLT. As for the random-effect factors, the models included individual intercepts and slopes for both session and intervention for the TDJT and the variable error of the TLT. In contrast, for the 2PDT, TMT and the constant error magnitude of the TLT, only intervention was included as a random-effect factor, since models including session failed to converge and displayed poorer fit (see electronic supplementary material, table S2). For the TLT, given that phalanx and position had more than two levels, type II Wald χ^2^ tests were performed on the LMM-fitted data to examine main effects and interactions involving these factors. When significant main effects or interactions were found, *post-hoc* pairwise comparisons between estimated marginal means computed from the LMM (R package emmeans) were conducted with alpha levels Bonferroni corrected for the number of tests performed (α_Bonf_). Effect sizes were computed as standardized mean differences (Cohen’s *d*-like), using the residual standard deviation estimated from the LMM [36]. Additionally, the post and pre slopes and their 95% confidence intervals (CIs) were derived from the LMMs for both RSS and sham conditions. A significant effect of intervention (sham or RSS) was indicated by a 95% CI that did not include zero, while the absence of an intervention effect was indicated by a 95% CI that included zero.

In the TLT data, a generalization of two-way ANOVAs for circular data (Harrison–Kanji test) was conducted for the constant error direction with the factors intervention and session, with alpha levels Bonferroni corrected for the number of tests performed (α_Bonf_ = 0.006). This test was performed using the CircStat toolbox for MATLAB [37]. For all tasks, Bayesian *t*-tests were conducted for comparisons of interest (pre versus post of each intervention), with a Cauchy prior width set to 0.707 (default). We reported the corresponding Bayes factors (BF_10_), showing the relative support for the alternative hypothesis, using the threshold of BF_10_< 1/3 as sufficient evidence in support of the null hypothesis [38]. In addition to these intervention-related tests, we examined intervention-unrelated perceptual biases. To determine the localization biases in the ulnar-radial (*x*) and proximo-distal (*y*) axes, the *x* and *y* components of the ∆_JR_ vector were compared with zero (null bias) using one-sample two-tailed *t*-tests conducted on data averaged across interventions and sessions, given the lack of any significant interaction. Similarly, we examined the overall perceptual bias in the TDJT by testing the difference between the real and the judged distances using two one-sample two-tailed paired *t*-tests (one for each distance condition, 15 and 30 mm) and a two-tailed paired *t*‐test to compare the two conditions (see electronic supplementary material).

Except when specified otherwise, the threshold for statistical significance was set at *p* ≤ 0.05. Statistical analyses were performed through RStudio (v.2024.12.0). To allow comparability with previous works, complementary repeated-measures ANOVAs are reported in electronic supplementary material, table S3.

## 3. Results

### (a) Affecting tactile processes through repetitive somatosensory stimulation improves tactile perception

To assess whether RSS was effective, we evaluated its impact on rD2’s 2PDT threshold through an LMM. This analysis revealed that RSS was indeed effective, with 29 (out of 33) participants showing reduced thresholds after RSS (for detailed results, see electronic supplementary material, figure S2).

### (b) repetitive somatosensory stimulation impacts the *body image*: the stimulated finger is perceived as smaller

**Figure 2.**
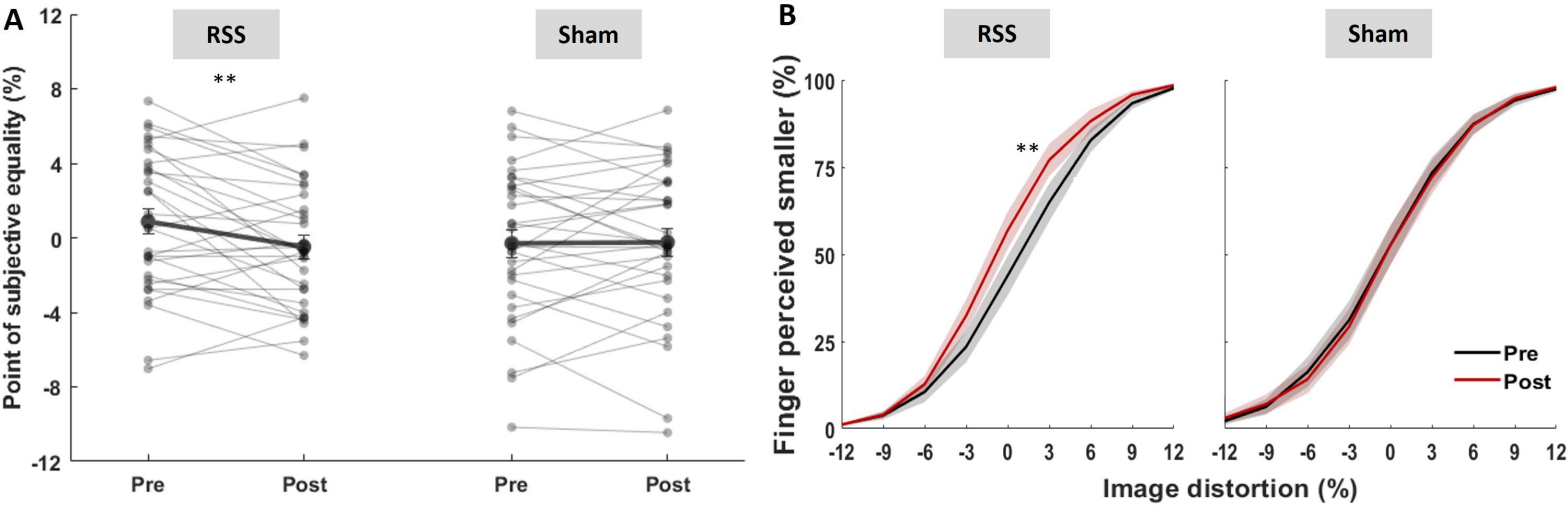
The stimulated finger is perceived as smaller after RSS but not after sham. (A) Individual (thin lines) and average (thick lines, ± s.e.m.) PSEs before and after intervention. (B) Mean psychometric curves of the TMT pre (black) and post (red) RSS or sham interventions (mean ± s.e.m.). **p < 0.01 (paired *t*-tests, α_Bonf_ = 0.025).

We then assessed whether RSS affected finger size perception (i.e. *body image*) of the stimulated finger with the TMT. An LMM revealed a significant intervention × session interaction (*t*_(60)_= −2.12, *p* = 0.038, *η*² = 0.07). *Post-hoc* pairwise comparisons showed that PSEs—expressed as the percentage of image distortion—were significantly smaller after RSS (*t*_(60)_= 2.89, *p* = 0.005, α_Bonf_ = 0.025, *d* = 0.73, BF_10_ = 4.76; figure 2A). On average, participants perceived their finger as −7.4 ± 4.1% (mean ± s.e.m.) smaller than it was before RSS, with 19 of them (out of 31) showing reduced PSEs after RSS. In contrast, no significant change was observed after the sham intervention (*t*_(60)_ = −0.11, *p* = 0.912, *d* = −0.03, BF_10_ = 0.19). Post and pre slopes derived from the LMM exhibited 95% CI that did not overlap with zero in the RSS condition (−2.30, −0.42), while they did in the sham condition (−0.89, 0.99; figure 2A). No significant main effect or interaction involving session and intervention was found on psychometric curves’ slopes (all *t*_(30–60)_ ≤ 0.72, *p* ≥ 0.47, *η*² ≤ 0.009; figure 2B).

### (c) Repetitive somatosensory stimulation does not seem to affect the *body model* and *superficial schema* of the stimulated finger

**Figure 3.**
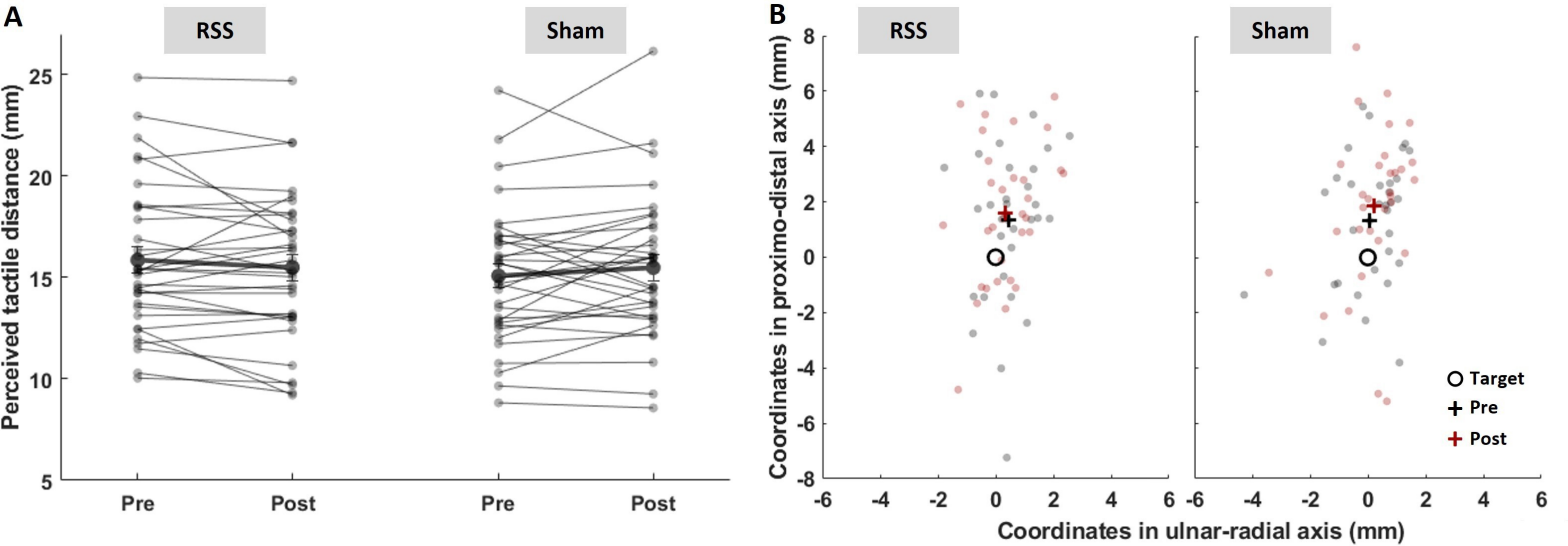
RSS does not seem to alter the perceived tactile distance or tactile localization. (A) Individual (thin lines) and average (thick lines, ± s.e.m.) perceived tactile distances before and after intervention. The data represented are averaged across the two positions (tip and base) and distances (15 and 30 mm). (B) Individual (dots) and average (crosses) judged positions relative to the target point (circle) pre (black) and post (red) RSS or sham interventions. The data represented are averaged across the nine points.

The TDJT was used to assess any effect of RSS on the *body model* and *superficial schema*. An LMM showed no significant main effect or interaction involving the factors intervention and session (all *t*_(59–5168)_ ≤ 1.41, *p* ≥ 0.159, *η*² ≤ 0.001; figure 3A). Bayesian *t*-tests revealed BFs close to 1/3 (sham: BF_10_ = 0.39; RSS: BF_10_ = 0.42). Additionally, post-pre slopes derived from the LMM exhibited 95% CI that overlapped with zero for both RSS (−0.87, 0.14) and sham (−0.11, 0.91) interventions. Similar results were obtained without outliers (*n* = 31; electronic supplementary material, tables S4 and S5).

The TLT was used to assess the *superficial schema*. Localization performance was compared with the actual target position for each of the nine locations along the finger. Their *x* and *y* coordinates were used to compute two localization error estimates: the constant error (magnitude and direction) and the variable error (see Methods for details). The type II Wald χ^2^ tests run on the LMM fitted with the data revealed no significant main effects or interactions involving the factors intervention and session for either the constant error magnitude (all *χ^2^*_(1–4)_ ≤ 3.27, *p* ≥ 0.195, *η*² ≤ 0.35) or the variable error (all *χ²*_(1–4)_ ≤ 3.70, *p* ≥ 0.094, *η*² < 0.001; figure 3B; electronic supplementary material, tables S4, S5). For the constant error magnitude, post-pre slopes derived from the LMM exhibited 95% CI that overlapped with zero for both RSS (−0.50, 0.32) and sham (−0.14, 0.68) interventions. For the variable error, these 95% CI were (−17.70, 13.60) in the RSS condition and (−33.80, −2.60) in the sham condition. The Harrison–Kanji tests conducted on the constant error direction measured at the nine locations revealed no significant main effects or interaction involving the factors intervention and session (all *Χ² *≤ 6.316, all *p* ≥ 0.043, α_Bonf_ = 0.006; electronic supplementary material, table S6). Bayesian *t*-tests revealed moderate evidence against an effect of RSS for the constant error magnitude (BF_10_ = 0.21) and variable error (BF_10_ = 0.19) and less conclusive ones regarding the effect of RSS on the constant error direction (BF_10_ = 0.43), and of sham (magnitude: BF_10_ = 0.42; direction: BF_10_ = 1.77; variable error: BF_10_ = 1.25). The absence of the RSS effect in all three measures (constant error magnitude, direction and variable error), as analysed both with type II Wald *χ*^2^ tests and 95% CI, was confirmed in the analysis without outliers. However, the effect of sham on the post-pre slopes 95% CI of the variable error (−21.1, 4.40) was no longer significant, suggesting it should be considered as not reliable. Additionally, a main effect of session on the variable error (*χ²*_(1)_ = 3.88, *p* = 0.049, *η²* = 0.001) was found (electronic supplementary material, tables S4, S5).

Overall, the intervention-related results showed that RSS, which was effective in reducing the 2PDT threshold at the stimulated finger, significantly affected only one of the three measures we assessed, namely that considered to tap into the *body image*. No significant correlation was found between changes in TMT thresholds and changes in 2PDT thresholds (*r* = 0.16, *p* = 0.396).

### (d) A systematic pattern of localization bias along the finger unrelated to repetitive somatosensory stimulation

**Figure 4.**
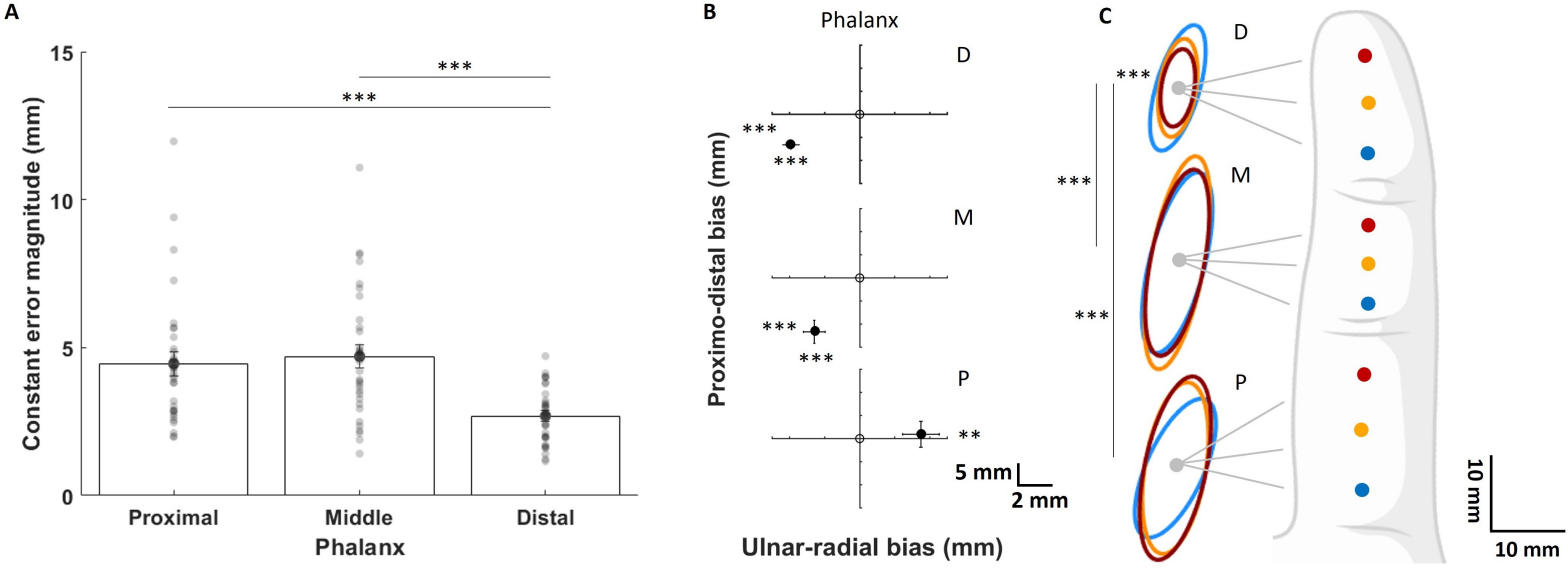
The TLT uncovered a pattern of perceptual bias along the finger. (A) Mean (± s.e.m.) constant error magnitudes in the three phalanges. Errors were averaged across the three locations within each phalanx as they did not differ significantly (all *F*_(2,120)_ ≤ 1.19, *p* ≥ 0.31). (B) Mean (± s.e.m.) difference between judged and target locations in ulnar-radial (*x*) and proximo-distal axis (*y*) at each phalanx. (C) Ellipses corresponding to the variable error for each of the nine target points. In the three panels, data are averaged across interventions and sessions since those were not different (see main text). P: proximal; M: middle; D: distal phalanges. ***p < 0.001; **p < 0.01. (A: paired *t*-tests, α_Bonf_ = 0.017; B: one-sample *t*-tests against zero, α_Bonf_ = 0.017; C: paired *t*-tests, α_Bonf_ = 0.0014).

Besides intervention-related effects, the type II Wald *χ*^2^ tests run on the LMM fitted with data of the constant error magnitudes (all interventions and sessions) also revealed a significant difference between phalanges (*χ*^2^_(2)_ = 147.58, *p* < 0.001, *η²* = 0.96), arising from significantly shorter error magnitudes in the distal phalanx than in middle (*t*_(1020)_ = −11.13, *p* < 0.001, α_Bonf_ = 0.017, *d* = −0.82) and proximal (*t*_(1020)_ = −9.79, *p* < 0.001, α_Bonf_ = 0.017, *d* = −0.72) phalanges (figure 4A). To further explore this error localization pattern, we then compared the *x* and *y* coordinates of participants’ mean localization relative to the target, using averaged data across interventions and sessions. One-sample two-tailed *t*-tests on the *x* component (i.e. lateral error) revealed a significant ulnar bias for the distal and middle phalanges (both *t*_(30)_ ≥ −4.18, both *p* < 0.001, α_Bonf_ = 0.017, both *d *≤ −0.75; figure 4B upper and middle panels), as well as a significant radial bias for the proximal phalanx (*t*_(30)_ = 3.34, *p* = 0.002, α_Bonf_ = 0.017, *d* = 0.60; figure 4B lower panel). In the proximo-distal axis, one-sample two-tailed *t*-tests on the *y* component revealed a significant proximal bias for the distal and middle phalanges (both *t*_(30)_ ≥ 4.68, both *p* < 0.001, α_Bonf_ = 0.017, both *d *≥ 1.306; figure 4B upper and middle panels). These results reveal a consistent bias in localization, with errors at the distal and middle phalanges directed towards the middle finger and the palm (proximo-ulnar bias), while errors at the proximal phalanx were directed towards the thumb (radial bias).

Similarly to the constant error, the type II Wald *χ*^2^ tests run on the LMM fitted with data of the variable error (all interventions and sessions) revealed a significant main effect of phalanx (*χ*^2^_(2)_ = 329.17, *p* < 0.001, *η²* = 0.05) arising from the areas of ellipses being significantly smaller at the distal than at the proximal (*t*_(1023)_ = −15.70, *p* < 0.001, α_Bonf_ = 0.017, *d* = −1.13) or middle (*t*_(1023)_ = −15.73, *p* < 0.001, α_Bonf_ = 0.017, *d* = −1.14) phalanges. A significant phalanx × position interaction was also observed (*χ*^2^_(4)_ = 31.27, *p* < 0.001, *η²* = 0.01), with a notable additional difference observed within the distal phalanx (see electronic supplementary material, table S7 for more details). Indeed, the most proximal ellipse (i.e. blue ellipse corresponding to the blue dot on phalanx D in figure 4C) was significantly bigger than the middle (yellow dot on phalanx D: *t*_(1023)_ = 2.99, *p* = 0.003, α_Bonf_ = 0.017, *d* = 0.37), and the distal one (red dot on phalanx D: *t*_(1023)_ = 4.51, *p* < 0.001, α_Bonf_ = 0.017, *d* = 0.56). Similar results were obtained without outliers (see electronic supplementary material, table S4) or when running the analysis on the baseline data only (i.e. before intervention; type II Wald *χ*^2^ tests: all *X² *≥ 14.49; all *p* ≤ 0.006; one-sample two-tailed *t*-tests: all *t *≥ 3.58; all *p* ≤ 0.001).

## 4. Discussion

The present study aimed to elucidate the link between some of the theorized MBRs and their supposedly shared somatosensory basis. To this aim, we investigated the effect of RSS—known to reduce the 2PDT thresholds of the stimulated finger by modulating SI activity [39]—on three tasks providing complementary measures of perceptual aspects of MBRs of the same finger. Following either RSS or sham intervention on the index finger, we assessed the *body image*, as probed through a TMT, the *body model*, as probed through a TDJT, and the *superficial schema*, as probed through the TLT, these tasks being based on the same multisensory (somatosensory and visual) information. We first ascertained RSS efficacy by replicating the expected finding of improved 2PDT performance at the index finger [34,39–41]. We then reported a reduction of perceived finger size following RSS. These results suggest that increasing somatosensory inputs shrinks the *body image*. Instead, RSS did not seem to significantly alter the *body model* or the *superficial schema*, though caution should be taken when interpreting these null results, as they were qualified by inconclusive BF values.

### (a) Increasing tactile inputs via repetitive somatosensory stimulation shrinks the *body image*

As recalled in the introduction, the *body image* is found to be either distorted or accurate, mainly depending upon the stimulation or tasks used. Distortions are mostly limited to cases where multisensory stimulations are used [15,16] or when they are measured through metric methods [13,14]. But when tackled by the tasks we selected in this study, the *body image* is most commonly reported to be relatively accurate [2,5,11]. Our findings corroborate this notion, as we found an average baseline distortion of only 0.3%. After RSS, whose efficacy was confirmed by the 2PDT improvement, participants perceived their finger as smaller than before. This suggests that increasing inputs through RSS shrunk the *body image*. This finding is consistent with the results of Gandevia & Phegan [23] and Ambron & Coslett [30], where the opposite modulation of inputs (i.e. reduction of inputs through anaesthesia) increased the perceived size of the anaesthetized body part. Altering tactile inputs thus seems to directly and bidirectionally impact the *body image*. This is consistent with the bidirectional alterations of the *body image* under the influence of multisensory stimulations (e.g. the rubber hand illusion; [42]). Our result is also consistent with a recent study [25] whereby high-frequency stimulation of 12 locations on the hand induced a reduction in the perceived size of the hand. Altogether, these findings suggest that the effects of RSS on the *body image* are likely robust.

One possible mechanism underlying the reduced *body image* of the finger may relate to the well-established effects of RSS on SI. By co-activating several skin receptive fields on the fingertip repeatedly for a protracted period of time, RSS was found to enlarge the stimulated finger’s representation in SI—increasing the neuronal resources available to process inputs—through long-term potentiation-like plasticity [34,39]. The *RSS-induced reduction* of *body image* through an increase of SI activity is coherent with the *anaesthesia-induced increase* of *body image* [23,30], possibly through an attenuation of SI activity [43].

### (b) Increasing tactile inputs via repetitive somatosensory stimulation does not seem to modify the *body model* and the *superficial schema*

Unlike the *body image*, the *body model* and *superficial schema* are thought to be distorted (though see 17). Previous work consistently reported an underestimation of the fingers’ length, together with a widening of the hand’s width [11,18]. Our results are in agreement with these distortions, as we found an underestimation of tactile distances, as well as a proximal bias in tactile localization for both the distal and middle phalanges and no proximo-distal bias in the proximal phalanx. Besides, our findings are coherent with well-established characteristics related to variations in mechanoreceptor density along the finger. Indeed, mechanoreceptor density increases towards the fingertip [44,45], enhancing spatial discrimination [44], perceived distances [46] and tactile localization accuracy [47]. Our findings replicate this, showing larger perceived distances and smaller localization errors at the distal phalanx (see electronic supplementary material).

In contrast to the *body image*, RSS did not significantly alter either the *body model* or the *superficial schema*. Nevertheless, because the non-significant difference across conditions and the inconclusive BFs do not allow for drawing firm conclusions about the absence of effect of RSS on these two MBRs, caution should be taken when interpreting these results. While acknowledging this note of caution, it can be reasonably concluded that RSS does not appear to affect the *body image* and the other two MBRs in a comparable way. This selective disruption of *body image,* but not the *body model*/*superficial schema,* mirrors previous findings. In anorexia nervosa [48] and fibromyalgia [49], the *body image* is altered, while the *superficial schema* [48] and *body model* [49] were found to be intact.

Besides tactile inputs, MBRs rely also on inputs from other modalities, such as vision and proprioception [19,20]. Manipulations of this sensory information have been reported to alter the *body model*, the *superficial schema* and an action-oriented MBR (the *body schema*). Taylor-Clarke *et al*. [31] found that visually magnifying the forearm and minifying the hand for 1 h decreased the well-established bias of perceiving greater distances on the finger. In other words, when the hand looks smaller, tactile distance is also perceived as smaller as compared with when the hand appearance is veridical. In the opposite direction, two other studies showed that when the hand looked larger, its maximum grip aperture to grasp an object was reduced [16] and tactile stimuli on that hand were localized more distally [50]—as if the hand was actually larger—as compared with when it looked veridical. Similarly, increased perceived tactile distance was observed when illusorily elongating the finger or the arm using either tendon stimulation [32] or a multisensory audio-tactile task [22]. These findings concur in showing that altering the perceived size of a body part (but not necessarily its *body image*) can affect the perceived distance between two points (*body model*) and the perceived locations of single points (*superficial schema*) applied on this body part, as well as its kinematics (*body schema*). Interestingly, similar visual and proprioceptive manipulations of a given body part were also reported to have an impact on body ownership [51] or on illusions (e.g. the rubber hand illusion: [15,42,52]) that both rely on the multisensory aspect of MBRs. This suggests that a change in the hand’s MBR can indeed be induced. However, it is worth noting that these manipulations consisted essentially of multisensory illusions, making them less comparable to tactile input alterations as implemented in the present work or following anaesthesia. Altogether, we suggest that the *body model* and *superficial schema* may be less vulnerable to tactile manipulations than the *body image*. This may reflect either: (i) their relative ‘immunity’ to changes in tactile inputs or (ii) their higher susceptibility to correction. Indeed, the *body model* and *superficial schema* may generally be more rigid (i.e. their distortions seem less susceptible to change) than the *body image*. As shown by Longo & Haggard [11,12] and Mancini *et al*. [18], the distortions of the *body model* and *superficial schema* are still found in different finger postures and hand orientations. As for the potential correction, its mechanisms could be of a different nature. Given that MBRs are multisensory [19], one possibility is that the altered tactile information could be compensated for by the intact proprioceptive and visual information. Additionally, recent work [10,53,54] suggests that localizing tactile stimuli on the skin could require a correction factor. In this respect, the RSS-induced change in SI information received by the *superficial schema* and *body model* might have been corrected by this factor. This factor might apply only to MBRs that are distorted and more action control related [53] and not (or less so) to MBRs that are accurate and more perception related, such as the *body image* [55].

Alternatively, this differential effect of tactile input changes on MBRs may stem from their potential dependence on partially different neural substrates. While there is no consensus about the neural bases of the different MBRs, they were found to rely on wide networks involving multiple cortical areas, with a notably large contribution of areas in the posterior parietal cortex [1,3]. Within the parietal cortex, while all three MBRs were found to involve the inferior parietal lobule [3,56], the *body model* and *superficial schema* seem to further rely on the superior parietal lobule and the temporoparietal junction [3,57,58]. Besides, whereas no evidence suggests the involvement of extra-parietal areas for the *body model* and the *superficial schema*, the *body image* seems to additionally rely on non-parietal regions, namely the extrastriate body area [59,60], the anterior insula [60] and other areas in the frontal lobe [61,62]. In this view, we speculate that RSS could have affected differently the areas both within and outside the parietal cortex.

### (c) Changes in *body image* are not linked to changes in perceptual thresholds

The lack of correlation between the *body image* and 2PDT threshold changes suggests that they are not linearly related, though their concomitant reduction is coherent with the higher 2PDT thresholds found in patients with enlarged *body image* (i.e. anorexia nervosa and complex regional pain syndrome; [63,64]), as compared with healthy populations. Yet, some studies reported an inverse relationship between such changes, with: (i) reduced 2PDT thresholds and an increased *body image* size following anaesthesia [30] or (ii) increased 2PDT thresholds and reduced *body image* size following tendon vibration [65]. Thus, the direction of change of the 2PDT threshold does not seem to depend on the direction of change of the *body image* size.

### (d) A novel pattern of localization bias along the finger

Besides replicating known distortions in MBRs and providing evidence for their differential sensitivity to modulation and reliance on tactile inputs, our results also bring new insights regarding MBRs. Intriguingly, we observed a specific pattern of ulnar-radial localization bias across phalanges, with the localization biased towards the thumb in the proximal phalanx and towards the middle finger distally. Although localization within a single finger has rarely been investigated [66], studies assessing landmark [67] and tactile [68] localization at the whole-hand scale, targeting the tips and bases of fingers did not report such an ulnar-radial bias. This discrepancy may arise from the fact that they targeted the fingertip and the skin crease at the base of the finger, which can be considered as ‘landmarks’, while we targeted points away from creases/joints, equally distributed along the finger. It could also be due to postural differences, as our study used a ‘natural’ posture with relaxed fingers, while previous studies used abducted fingers, which are linked to a wider hand representation [69]. Nevertheless, the radial bias of the proximal phalanx we newly report here seems in keeping with the radial bias found on the palm [70]. Our findings may reflect a bias towards adjacent fingers, at locations where informative tactile (co-)stimulation across fingertips is more likely to occur due to postural and movement synergies. Indeed, in a natural hand posture, the proximal phalanx of the index finger contacts both the middle finger and the thumb while the other two phalanges contact only the middle finger. This finding raises new questions about the role of tactile ‘synergies’ in the distortions observed at the level of the *superficial schema*.

### (e) Updating the theoretical framework of mental body representations

**Figure 5.**
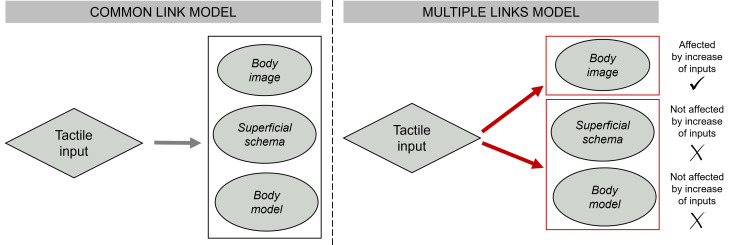
Updating the model of MBR–tactile input relationship. There was no known distinction between the three MBRs’ relationship to tactile input. Showing that RSS selectively affects the *body image*, we propose a model revision that distinguishes it from the other MBRs.

These findings help revise the model of MBRs, in particular with respect to their relationships with tactile inputs (figure 5). Indeed, the relationship between tactile inputs and the *body image* appears different from those linking them to the *superficial schema* and *body model*. While RSS is known to affect SI—nourishing the parietal cortex that appears to be crucial for MBRs [1,3]—this effect alone cannot account for the whole pattern of results observed in this study. The way SI exerts its modulatory effects differently on MBRs may either depend upon the MBRs relating to SI in a different way (i.e. the *body model* and *superficial schema* may be less susceptible to change in inputs from SI), and/or upon different cortical areas underpinning the *body imag*e and the other two MBRs. While future work will help disentangle these alternatives, our study provides novel evidence on the neglected link between MBRs and somatosensory processes and paves the way for novel clinical applications for the treatment of pathological MBR conditions.

## 5. Conclusion

We provide evidence that converges with previous work in indicating that the *body image* is bi-directionally susceptible to changes following a temporary modulation of tactile inputs. Our findings also indicate that the tested MBRs, even if all nourished by tactile afferents through SI, are not affected in the same way by increasing tactile information. We suggest that the *body model* and *superficial schema* may be more rigid and less (or more indirectly) affected by modulation of tactile inputs. Importantly, this study provides a proof of concept that a simple, non-invasive and effortless tactile stimulation can alter the *body image* in the direction of a reduction of the perceived body size, which could translate into rehabilitative strategies to help treat *body image* disturbance, frequently occurring in eating disorders (anorexia nervosa, bulimia).

## Data Availability

The data and codes underlying this study are openly available in Dryad at [71]. Supplementary material is available online [72].
